# Supplementary material for: Electrical noise spectroscopy of magnons in a quantum Hall ferromagnet
Source: Nat Commun. 2024 Jun 12;15:4998. doi: 10.1038/s41467-024-49446-z (PMC11169481; doi:10.1038/s41467-024-49446-z)
Supplement: Supplementary file 1 — Supplementary Information [file 41467_2024_49446_MOESM1_ESM.pdf]

Supplementary information: Electrical noise spectroscopy of magnons in a quantum Hall ferromagnet

Ravi Kumar[1,*], Saurabh Kumar Srivastav[1,*], Ujjal Roy[1,*], Jinhong Park[2,3,*], Christian Spånslätt[4], K. Watanabe[5], T. Taniguchi[5], Yuval Gefen[6], Alexander D. Mirlin[2,3], and Anindya Das[1†]

[1]*Department of Physics, Indian Institute of Science, Bangalore, 560012, India.*

[2]*Institute for Quantum Materials and Technologies, Karlsruhe Institute of Technology, 76021 Karlsruhe, Germany.*

[3]*Institut für Theorie der Kondensierten Materie, Karlsruhe Institute of Technology, 76128 Karlsruhe, Germany.*

[4]*Department of Microtechnology and Nanoscience (MC2), Chalmers University of Technology, S-412 96 Göteborg, Sweden.*

[5]*National Institute of Material Science, 1-1 Namiki, Tsukuba 305-0044, Japan.*

[6]*Department of Condensed Matter Physics, Weizmann Institute of Science, Rehovot 76100, Israel.*

[*]These authors contributed equally: Ravi Kumar, Saurabh Kumar Srivastav, Ujjal Roy, Jinhong Park

[†]anindya@iisc.ac.in

This supplementary information contains the following details:

1. Device fabrication, characterization, and noise measurement setup

2. Noise and non-local resistance at $\nu = 1$ for various magnetic fields

3. Threshold voltage of magnon detection from noise and non-local resistance measurements

4. Temperature dependence of the non-local resistance

5. Contribution of phonons

6. Response of the bilayer graphene device

7. Noise of the bilayer graphene device

8. Gain and electron temperature estimation

9. Theoretical model

- **9. 1. Key assumptions and hierarchy of energy scales**
- **9. 2. Model, electrical current, and noise**
- **9. 3. Current and noise in a single tunnel junction model**
- **9. 4. Current and noise in a line junction model**
- **9. 5. Noise generated in a line junction**
- **9. 6. Voltage dependence of equilibration length, overall behavior of the noise, and comparison to experimental data**

Section S1: Device fabrication, characterization, and noise measurement setup

Utilizing the dry transfer pick-up approach[1,2], we fabricated an encapsulated device consisting of a heterostructure involving hBN (hexagonal boron nitride), single-layer graphene (SLG), and graphite layers. The procedure for creating this heterostructure comprised mechanical exfoliation of hBN and graphite crystals onto an oxidized silicon wafer through the widely employed scotch tape method. Initially, a layer of hBN, with a thickness of approximately 25-30 nm, was picked up at a temperature of 90^oC. This was achieved using a Poly-Bisphenol-A-Carbonate (PC) coated Polydimethylsiloxane (PDMS) stamp positioned on a glass slide, attached to a home-built micromanipulator. The hBN flake was then aligned over the previously exfoliated SLG layer, which was similarly picked up at 90^oC. The subsequent step involved picking up the bottom hBN layer of similar thickness. Following the same process as above, this bottom hBN was picked up utilizing the previously acquired hBN/SLG assembly. Subsequent to this, the hBN/SLG/hBN heterostructure was employed to pick up the graphite flake. Ultimately, this resulting heterostructure (hBN/SLG/hBN/graphite) was placed on top of a 285 nm thick oxidized silicon wafer at a temperature of 180^oC. To remove the residues of PC, this final stack was cleaned in chloroform (CHCl3) overnight, followed by cleaning in acetone and isopropyl alcohol (IPA). After this, Poly-methyl-methacrylate (PMMA) photoresist was coated on this heterostructure to define the contact regions using electron beam lithography (EBL). Apart from the conventional contacts, we defined a region of $\sim 6\mu$m^2 area in the middle of SLG flake, which acts as a floating metallic reservoir upon edge contact metallization. After EBL, reactive ion etching (with a mixture of CHF3 and O2 gas with a flow rate of 40 sccm and 4 sccm, respectively, at 25^oC with RF power of 60W) was used to define the edge contact[3]. The etching time was optimized such that the bottom hBN did not etch completely in order to isolate the contacts from the bottom graphite flake, which was used as the back gate. Finally, thermal deposition of Cr/Pd/Au (3/12/60 nm) was done in an evaporator chamber having a base pressure of $\sim 1 \times 10^{-7}$ mbar. After deposition, a lift-off procedure was performed in hot acetone and IPA. The device's schematics and measurement setup are shown in Fig. 1(a) in the main text. The distance from the floating contact to the ground contacts is $\sim 5\mu$m, whereas the transverse contacts are placed at a distance of $\sim 2.5\mu$m.

All measurements were done in a cryo-free dilution refrigerator with a $\sim$ 20mK base temperature. The electrical conductance was measured using the standard lock-in technique, whereas the noise was measured using an LCR resonant circuit at resonance frequency $\sim$ 740kHz. A schematic of the noise measurement setup is shown in Fig. 1(a) in the main text. The device was mounted on a chip carrier, which was connected to the homemade cold finger fixed to the mixing chamber plate of the dilution refrigerator. The ground contact (CG) pins were directly shorted to the cold finger in order to achieve the cold ground. The sample was current- biased during the measurements. Noise signals were amplified with a homemade cryogenic voltage pre-amplifier, which was thermalized to the 4K plate of the dilution refrigerator. This pre-amplified signal was then amplified using a voltage amplifier placed at the top of the fridge at room temperature. After the second stage of amplification, the amplified signal was measured using a spectrum analyzer (N9010A). All

noise measurements were done using a bandwidth $\sim$ 30 kHz. The resonant L//C tank circuit was built using an inductor L of $\sim$ 365 μH made from a superconducting coil thermally anchored to the mixing chamber plate of the dilution refrigerator. A parallel capacitance C of $\sim$ 125 pF develops along the coaxial lines connecting the sample to the cryogenic pre-amplifier.

We used two devices: one made of single layer graphene and one made of bilayer graphene. The quantum Hall response of the single-layer graphene is shown in Supplementary Fig. 1(a). It can be seen that robust QH plateaus are observed at the magnetic field strength $B = 1$T. Supplementary Figs. 1(b) and (c) show the temperature dependence of the longitudinal resistance R_{XX} at $B = 1$T and 2T, respectively. The activation plots are shown in the insets, where we extract activation gaps of the $\nu = 1$ QH state. We estimate these gaps to $\sim$ 4K and 9K at $B = 1$T and $B = 2$T, respectively.

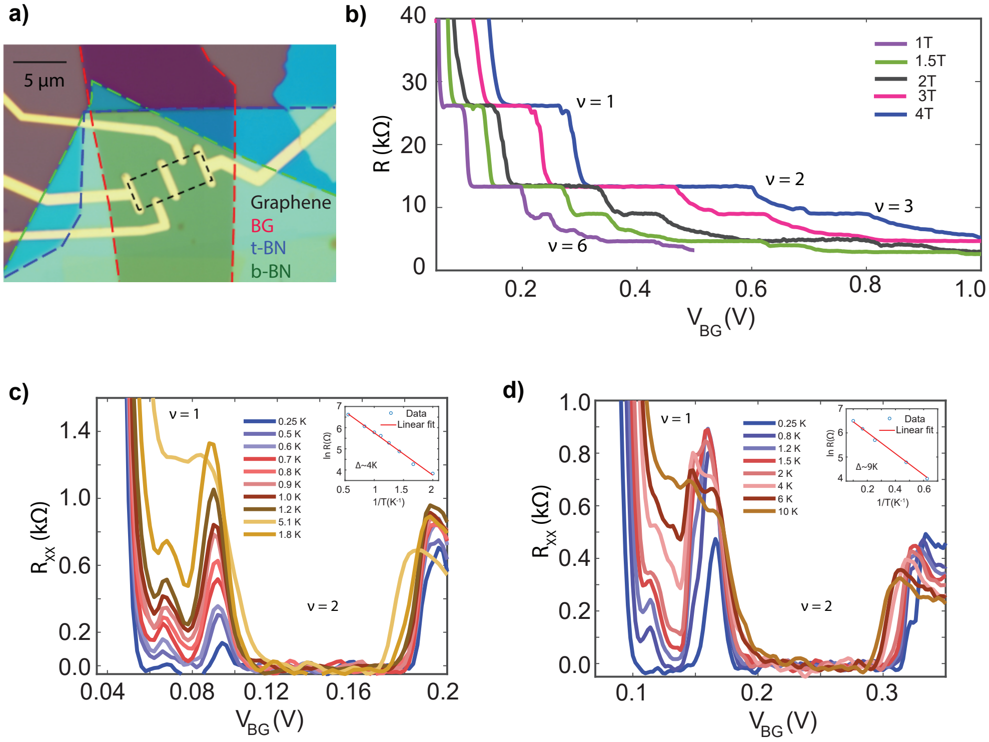

Supplementary Figure 1: Device image, QH response of the single layer graphene device and activation gap. (**a**) Optical image of the device. Graphene channel, back-gate, top hBN, and bottom hBN are outlined by dashed black, red, blue, and green lines, respectively. (**b**) Hall response of the single-layer device at various magnetic field strengths. Panels (**c**) and (**d**) shows the R_{XX} as a function of the gate voltage V_{BG} at various temperatures for $B = 1$T and $B = 2$T, respectively. The insets show the activation gaps for $\nu = 1$.

Section S2: Noise and non-local resistance at filling $\nu = 1$ for various magnetic fields

In this section, we present the non-local resistance and noise measured at several magnetic field strengths across the $\nu = 1$ QH plateaus of the single-layer graphene device. The measurement schemes are described in the main text and shown in Fig. 1(a) in the main manuscript. Supplementary Fig. 2 summarizes the non-local resistance and noise data, from which the threshold voltages (V_{th}) are extracted and shown in Fig. 2(f) in the main manuscript. How the threshold voltage was extracted is presented in the next section.

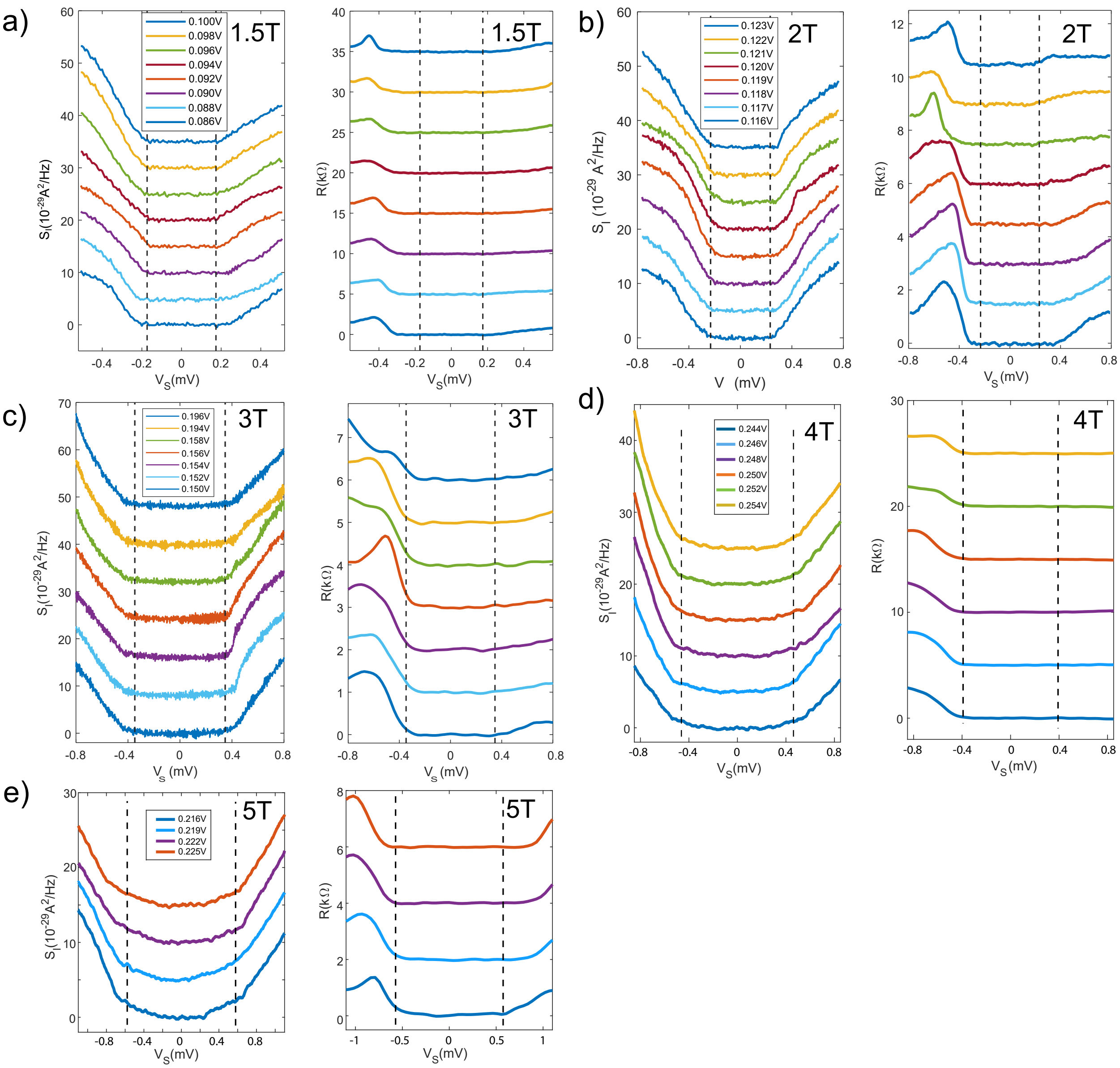

Supplementary Figure 2: Noise and non-local resistance. As described in the manuscript and shown in Fig. 1(a), the noise and the non-local resistance were measured. Panels (**a**), (**b**), (**c**), (**d**), and (**e**) show the measured noise (left column) and non-local resistance (right column) across the plateau of $\nu = 1$ at different magnetic fields ($B = 1.5$T, 2T, 3T, 4T, and 5T) for s single layer graphene device. The vertical dashed lines correspond to the Zeeman energy.

Section S3: Threshold voltage of magnon detection from noise and non-local resistance measurements

In this section, we describe how the threshold voltages (V_{th}) from the non-local resistance and noise data presented in Supplementary Fig. 2 were determined. Supplementary Fig. 3 shows one of the examples

at different magnetic fields for both the non-local resistance and noise data. For the threshold voltage, we calculate the *rms* value of the data, and a sudden change in its magnitude is marked as a threshold voltage, which is shown by the vertical black lines in Supplementary Fig. 3. At a given magnetic field, the V_{th} was extracted for several points across the plateau for the data set shown in Supplementary Fig. 2, and its mean value and standard deviation are shown in Fig. 2(f) in the main manuscript. For the noise data sets, we extract the V_{th} for both the positive and negative bias voltages and take its mean. However, for non-local resistance data sets, we extract the V_{th} for only the negative bias voltage side as the threshold voltage for the positive bias voltage side was not prominent. Note that for the noise data at $4T$ and 5T, V_{th} is calculated from the sudden change in the slope of noise, due to the presence of a finite background noise these magnetic fields, coming from phonons. The background noise at higher magnetic fields is shown in Supplementary Figure 5.

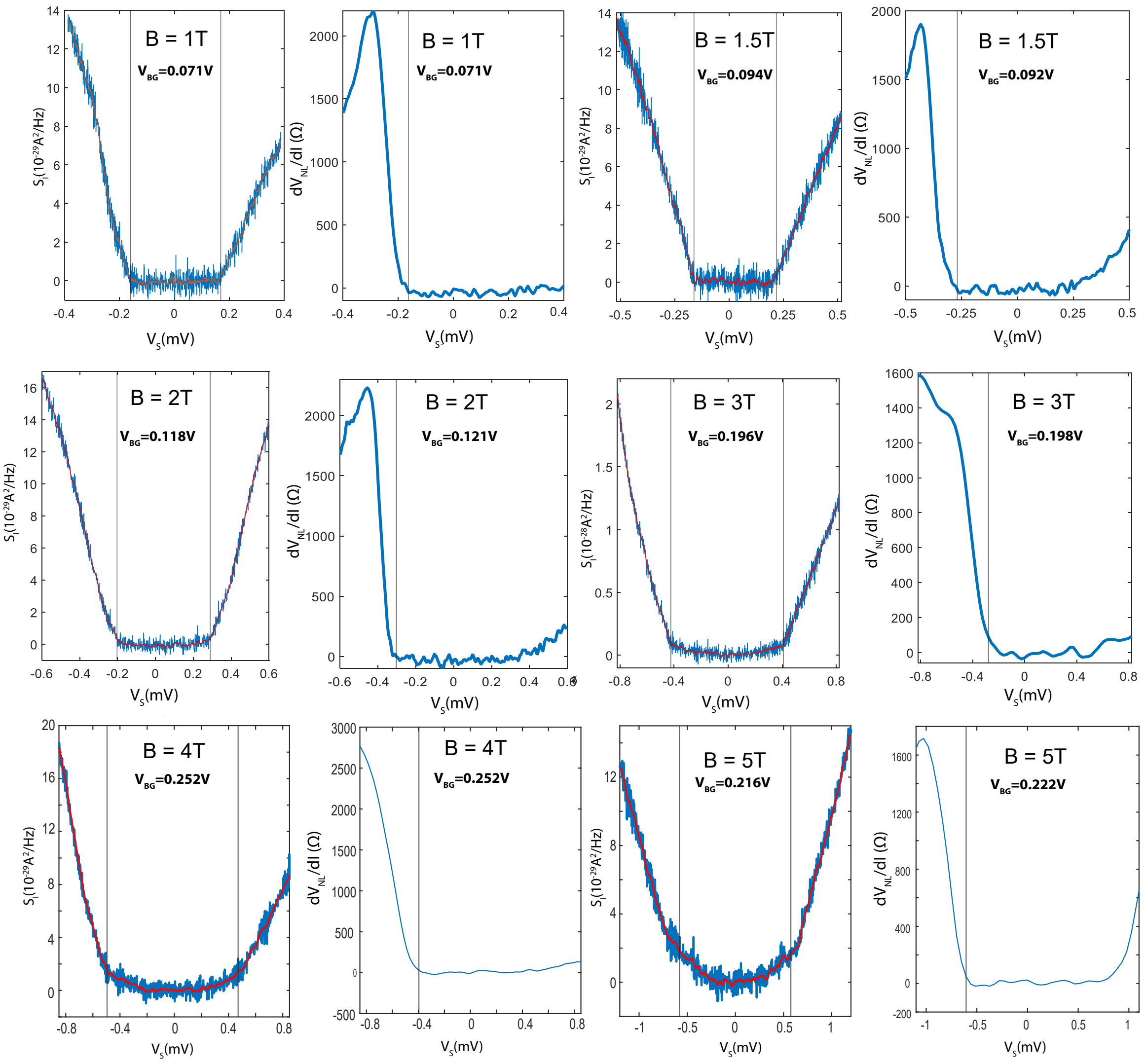

Supplementary Figure 3: Threshold voltage. To determine the threshold voltage from the noise data, we have taken a 10-point average of the raw data, shown by the red solid lines. We calculate the *rms* value of the average data, and a sudden change in its magnitude is marked as a threshold voltage, which is shown by the vertical black lines. We extract the threshold voltage for both the sign of bias voltages for the noise data and take its mean value. This exercise was done at several points of the plateau (points are shown in Supplementary Fig. 2), and its mean value and standard deviation for a given magnetic field are plotted in Fig. 2(f) in the main manuscript. A similar procedure was done for the non-local resistance data but only for the negative bias voltage side, as the transition for the positive bias voltage was hardly seen in most of the data. For noise data at 4T and 5T, the threshold voltage is determined by a sudden change in the slope.

Section S4: Temperature dependence of the non-local resistance

In this section, we present the temperature dependence of the non-local resistance measured at the $\nu = 1$ QH plateau for $B = 1$T in the single-layer graphene device. As can be seen in Supplementary Fig. 4, the threshold voltage remains higher than the Zeeman energy until 200mK, and with increasing temperature the data gets broadened, and V_{th} starts appearing at the vicinity of the Zeeman energy and even slightly lower than the Zeeman energy at sufficiently high temperature. A similar temperature-broadening effect was observed in the threshold voltage for the non-local resistance at $B = 3$T but at the base temperature ($T_0 =$ 20mK), as presented in Fig. 2(f) in the main manuscript. At a higher magnetic field ($B = 3$T), phonons play an important role since a larger current is required in order to generate magnons due to the higher Zeeman energy. Thus, the increased dissipation near **'A'** and **'E'** in Fig. 1(a) in the main manuscript is able to excite phonons even at the base temperature ($T_0 =$ 20mK). The contribution of phonons is discussed further in Sec. S5. Note that no non-local resistance was detected even at elevated temperature ($T_0 =$ 600mK) on the $\nu = 2$ QH plateau, which is expected due to its non-magnetic nature.

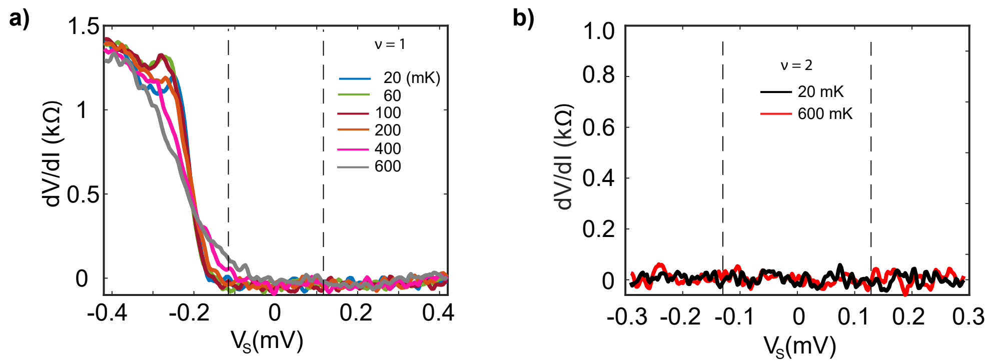

Supplementary Figure 4: Temperature dependence of the non-local resistance for (**a**) bulk filling $\nu = 1$, and (**b**) $\nu = 2$ at $B = 1$T. The non-local signal at $\nu = 1$ is almost the same up to 200 mK, and above 200 mK the data gets smoothed. No non-local signal was detected for $\nu = 2$ even at 600 mK as it is non-magnetic.

Section S5: Contribution of phonons

In this section, we present the contribution of phonons in our noise measurement for the single-layer graphene device. In order to know the contribution of phonons quantitatively, we measure the noise at the $\nu = 2$ non-magnetic QH plateau at several magnetic fields and at base temperature ($T_0 = 20$ mK). This is shown in Supplementary Fig. 5, where the x-axis is normalized with the Zeeman energy. Note that as described in Fig. 1(a) in the main manuscript, the application of a bias voltage at the source contact creates hot spots near **'A'** and **'E'**, which can excite phonons and can travel through the bulk of the device and in-

crease the temperature of the floating contact situated along the upstream direction, see Fig. 1(a) in the main manuscript. It can be seen from Supplementary Fig. 5 that at $B = 1$T and $B = 1.5$T, hardly any noise was detected. At $B = 2$T, a detectable noise from the phonon was measured, but its magnitude remains almost 6-7 times smaller than the noise measured for the $\nu = 1$ quantum Hall ferromagnetic phase, as shown in Fig. 2(e) in the main manuscript and Supplementary Fig. 2(b). At $B = 3$T, 4T, and 5T, significant noise was detected from phonons, but its magnitude remains smaller than the noise measured for the $\nu = 1$ quantum Hall ferromagnetic phase within our working bias range, as shown in Supplementary Fig. 2(c, d, e) for $B < 5T$.

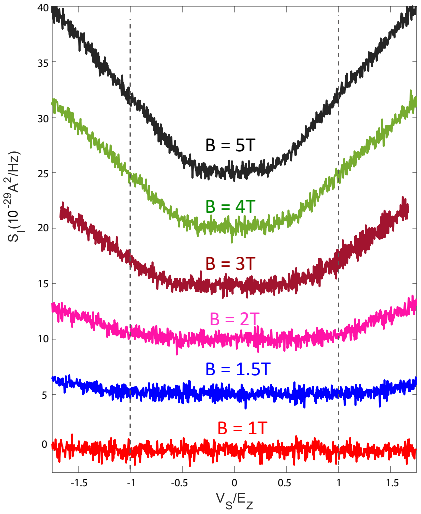

Supplementary Figure 5: Noise measured at $\nu = 2$. As described in the manuscript and shown in Fig. 1(a) in the main manuscript, the noise was measured along the upstream direction. Here, we show the measured noise at bulk filling $\nu = 2$ (which is non-magnetic) at different magnetic fields. For clarity, the data are vertically shifted by $5\times\ 10^{-29}\ A^2/Hz$.It can be seen that above $B = 3$T, 4T, and 5T significant noise due to phonons was detected. The bias voltages on the x-axes are normalized to the Zeeman energy, E_Z. The vertical dashed lines correspond to Zeeman energy. As mentioned in the main manuscript, at a higher magnetic field, a larger current is required to reach E_Z, and thus stronger hot spots are created (points **'A'** and **'E'** in Fig. 1(a) in the manuscript), which excite phonons. However, most of the data was analyzed at $B = 1 - 2$T, where almost no phonon contribution was detected.

Section S6: Response of the bilayer graphene device

So far, we have presented and discussed the data obtained from the single-layer graphene device. In this section, we present the non-local resistance and noise measurements for our second device, made with bilayer graphene. For the bilayer graphene device, local doping is independently controlled by SiO_2 gating

[shown by the gray filled region in Supplementary Fig. 6(a)], while the bulk filling of the device is controlled by the back graphite gate ["BG", shown as the sky blue filled region in Supplementary Fig. 6(a)]. Robust QH plateaus were observed at slightly higher magnetic fields, $B = 4$T, in contrast to $B = 1$T for the single-layer graphene device. This is shown in Supplementary Fig. 6(b), which depicts the resistance R measured at the source contact as a function of the graphite back gate at $B = 4$T for the two cases where the SiO_2 gate was tuned at $\nu = 2$ and $\nu = 4$, respectively. Though the quality of the bilayer graphene device was not as good as the single-layer graphene device, the measured noise remained qualitatively similar to the single-layer device, as presented in the next section.

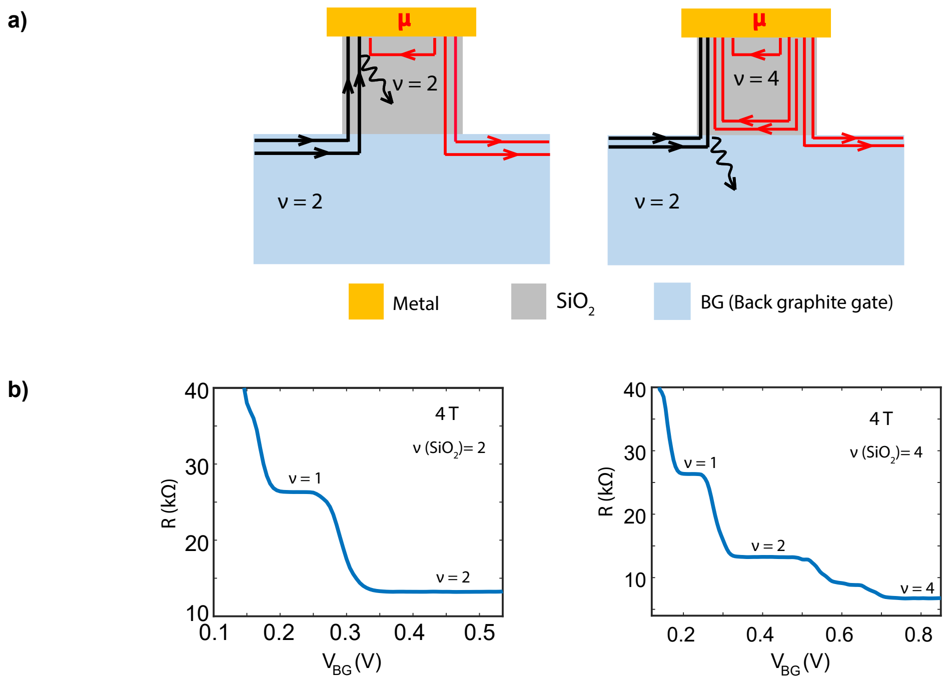

Supplementary Figure 6: Controlled local doping near the ohmic contact and response of the bilayer graphene device. For the bilayer graphene device, local doping is controlled by the SiO_2 (shown in gray) gating, while the device bulk filling is controlled by the back graphite gate (BG, shown in sky blue). (**a**) Left schematic: the bulk and local fillings were kept at $\nu = 2$. Right schematic: bulk and local fillings were kept at $\nu = 2$ and $\nu = 4$, respectively. The wavy lines indicate the magnon generation region for two different filling configurations. The corresponding QH responses as a function of the graphite back gate of the device are shown in panel (**b**) at $B = 4$T. For this measurement, the voltage was measured at the same source (current injected) contact.

Section S7: Noise of the bilayer graphene device

In this section, we will discuss the noise measured for the bilayer graphene device. For bilayer graphene, $\nu = 2$ is a quantum Hall ferromagnet whereas $\nu = 4$ is non-magnetic[4,5] as shown schematically in Supplementary Fig. 7(b). The scheme for the noise measurement is shown in Supplementary Fig. 7(a) and discussed in details in the figure caption. Supplementary Fig. 7(c) shows a 2D color plot of the measured noise at $B = 4$T of the device, where the bulk filling is set to $\nu = 2$ while the filling near the contact is kept at $\nu = 4$ (see Supplementary Fig. 6(a), right panel). The vertical dashed lines in Supplementary Fig. 7(c) correspond to the Zeeman energy and the solid magenta line corresponds to one of the line cuts. It can be seen that noise increases almost linearly for bias voltages above the Zeeman energy. However, it should be noted that there is finite noise measured even inside the Zeeman energy. This is not surprising as the measurement for the bilayer graphene device was done at $B = 4$T, and the phonon contribution was not negligible: As shown in Supplementary Fig. 7(d), there is finite noise even for the non-magnetic $\nu = 4$ QH state.

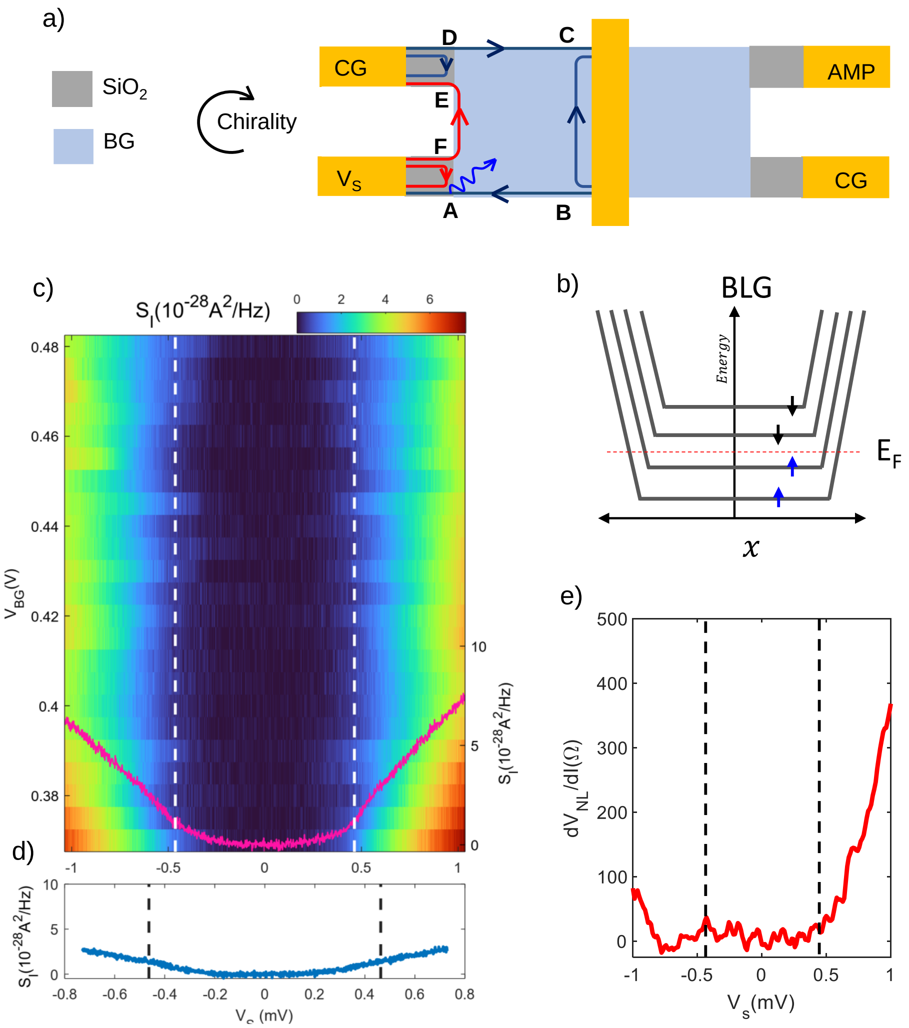

Supplementary Figure 7: Measured noise at $\nu = 2$. For bilayer graphene, $\nu = 2$ is a quantum Hall ferromagnet whereas $\nu = 4$ is non-magnetic[4,5] as shown in (**b**). (**a**) Device schematic for noise measurement. The V_S, CG, and AMP represent the voltage source, cold ground, and amplifier, respectively. The magnons are generated at points **'A'** and **'E'** for negative and positive bias voltages, respectively. In the schematic, it is shown only for negative bias voltage (by the wiggly line with an arrow). Note that in the schematic, each line with arrow corresponds to two edge modes for bilayer graphene (for bulk filling $\nu = 2$); for simplicity, we have shown a single line. The absorption at points **'B'** and **'D'** contribute to the noise and non-local resistance. (**c**) The 2D color plot of the measured noise (as described in Fig. 1(a) of the manuscript) at $B = 4$T of the device, where the bulk filling is set to $\nu = 2$ while the filling near the contact is kept at $\nu = 4$. The vertical dashed lines correspond to the Zeeman energy. The solid magenta line corresponds to one of the cut lines. It can be seen that noise increases almost linearly above the Zeeman energy. (**d**) Noise as a function of the bias voltage when both the filling of the bulk and contact are kept at $\nu = 4$. As expected, there is some contribution of noise from phonons. (**e**) Non-local resistance as a function of bias voltage. It can be seen that for negative bias voltage, the non-local signal is weaker as the distance from magnon generation point **'A'** to the absorption points **'B'** and **'D'** are almost equal.

Section S8: Gain and electron temperature estimation

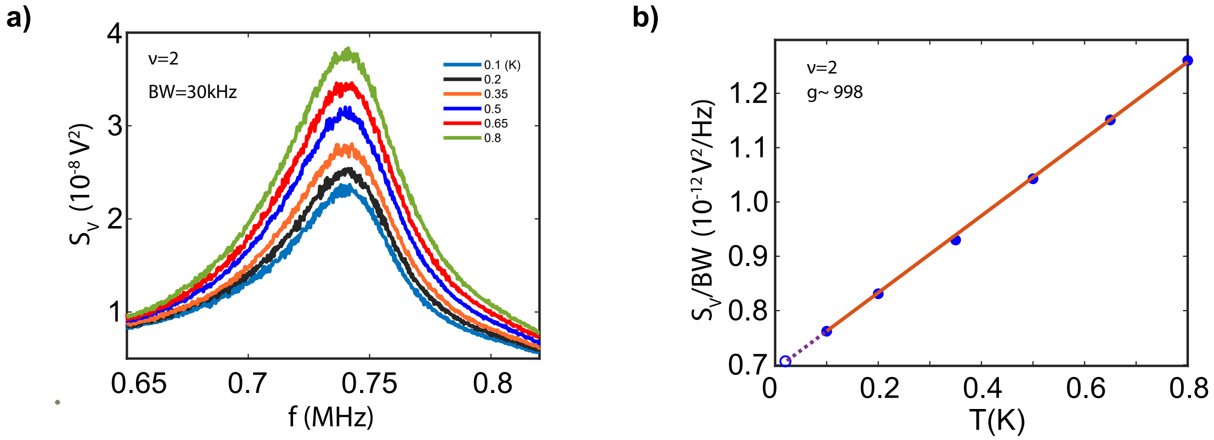

Supplementary Figure 8: Gain and electron temperature estimation at $\nu = 2$. (**a**) The voltage noise S_V [see Eq. (S1)] measured by a spectrum analyzer is plotted as a function of the frequency at different bath temperatures for $\nu = 2$. From this plot, the resonance frequency of the tank circuit was found to be ∼740 kHz. (**b**) Blue solid circles represent the noise S_V divided by bandwidth (BW) at the resonance frequency as a function of the bath temperature. The solid red line is a linear fit to the data from 0.1 K to 0.8 K and the dashed line is the linear extrapolation below 0.1 K. Using Eq. (S2) and the slope of this linear fit, the gain g was found to be equal to ∼998.

In our analysis of the noise data, it is crucial to know the gain of the amplifier chain and the electron temperature (T_e) of the system. In this section, we describe how we measure these two quantities. The electron temperature does not need to be the same as the bath temperature (T_{bath}) of the system (temperature of the MC plate in our experiment). We calculate precisely the gain and T_e by measuring thermal noise at zero current bias.

We have estimated the gain of the amplification chain and the electron temperature from temperature-dependent Johnson-Nyquist noise (thermal noise). At zero impinging current, the equilibrium voltage noise spectrum is given by

$$S_V = g^2(4k_BTR + V_n^2 + i_n^2R^2)BW\,, \tag{S1}$$

where g is the total gain of amplification chain, k_B the Boltzmann constant, T the bath temperature (temperature of mixing chamber (MC) plate), R is the resistance of quantum hall state, V_n^2 and i_n^2 are the intrinsic voltage and current noise of the amplifier, and BW is the frequency bandwidth. The first term, $4k_BTR$ corresponds to the thermal noise. At a quantum Hall plateau, any change in bath temperature will only affect the first term in Eq. (S1), while all other terms are independent of temperature. If one plots the $\frac{S_V}{BW}$ as a function of temperature, the slope of the linear curve will be equal to $4g^2k_BR$. Since at the quantum Hall

plateau, the resistance R is exactly known, one can easily extract the gain of the amplification chain from the slope and the intrinsic noise of the amplifier from the intercept. The gain is found using the following equation:

$$g = \sqrt{\Big(\frac{\partial\big(\frac{S_V}{BW}\big)}{\partial T}\Big)\Big(\frac{1}{4k_BR}\Big)}\,, \tag{S2}$$

where $\Big(\frac{\partial\big(\frac{S_V}{BW}\big)}{\partial T}\Big)$ is the slope of the linear fit. The implementation of this procedure is shown in Supplementary Fig. 8 for $\nu = 2$.

The noise spectrum (S_V) at zero impinging current measured on the $\nu = 2$ plateau at different bath temperatures is shown as a function of frequency in Supplementary Fig. 8(a). The S_V value at the resonance frequency, divided by BW, is plotted as a function of bath temperature in Supplementary Fig. 8(b), where the red solid line is the linear fit to the data in the temperature range from 0.1K to 0.8K. From the slope, we extract the gain, which is found to be ~ 998. Note that we do not use the base temperature data for the fitting in Supplementary Fig. 8(b), because the electron temperature (T_e) could be different from the base temperature.

As the gain is known, one can calculate the $(V_n^2 + i_n^2R^2)$ from the intercept of the linear fitting of S_v/BW vs temperature. Now from the known value of the measured noise at the base temperature, the corresponding electron temperature (T_e) can be found directly using the following equation:

$$T_e = \frac{\Bigg(\bigg(\frac{S_V}{g^2BW}\bigg) - (V_n^2 + i_n^2R^2)\Bigg)}{4k_BR}\,. \tag{S3}$$

The measured value of noise for $\nu = 2$ at base temperature corresponds to $T_e = 23\,\text{mK}$, which is consistent with the electron temperature measured in our previous work[6,7]. The fact that T_e is very close to the bath temperature can be also seen directly from Supplementary Fig. 8(b): The $T_{bath} = 20\,\text{mK}$ data point (blue open circle) is located almost on the dashed line representing the extrapolation of the linear fit into the region below $0.1\,\text{K}$.

Section S9: Theoretical model

In this section, we calculate the electrical current and noise generated in the magnon absorption regions. We perform this calculation with two different models describing the magnon generation and absorption regions (**’A’**, **’B’**, **’C’**, **’D’**, **’E’**, and **’F’**, see Fig. 1(a) in the main text): (i) a single tunnel junction model and (ii) a line junction model. In the single tunnel junction model, we assume that the equilibration between edge channels and magnons in those regions is only partial. In other words, the characteristic equilibration length scale ℓ_{eq} is much larger than the physical length L of the regions. In contrast, the line junction model assumes $\ell_{\text{eq}} < L$, i.e., full equilibration. As we show below, the two models yield distinct electric current and noise characteristics. We further show that the first model is applicable in a range of bias voltages $|eV_S|$ sufficiently close to E_Z, while the second model is justified for larger $|eV_S|$. Finally, we compare these theoretical predictions with our experimental results.

A word of caution concerning the terminology is in order at this point. Strictly speaking, the length ℓ_{eq} corresponds to a pre-equilibration. The true equilibration at low temperatures $T \ll E_Z$ takes place at an exponentially large length and is thus not relevant experimentally. We discuss this point in more detail below in subsection S9.4.

This theoretical section is organized as follows. In subsection S9. 1, we establish the key assumptions of the calculations and the hierarchy of involved energy scales in the system. In subsections S9. 2 and S9. 3, we calculate the electrical current and noise in the single tunnel junction model. The following subsection S9. 4 presents the line junction model and its associated current and noise characteristics. Technical details of the calculation of noise are presented in S9. 5. In the last subsection S9. 6, we calculate the equilibration length, determine ranges of validity of both models, summarize our theoretical predictions, and compare them with the experimental data.

S9.1. Key assumptions and hierarchy of energy scales. Before diving into the actual calculation, we first establish the hierarchy of involved energy scales in the system. The relevant energy scales are the bias energy $|eV_S|$, the Zeeman energy E_Z (which is the minimal energy of a magnon), the total inter-edge channel electron tunneling rate Γ in the absorption regions (or, equivalently, the total magnon absorption rate), the thermal energy k_BT_0, and the level spacing δE of the magnonic spectrum. In our calculations, we assume the following hierarchy:

$$\delta E < k_BT_0 \ll E_Z < |eV_S|. \tag{S4}$$

From the condition $k_BT_0 \ll E_Z$, we can neglect any generation of magnons by thermal excitations, so that the magnon generation is almost entirely due to tunneling of electrons between edge channels. In the regime $|eV_S| < E_Z$, this tunneling process, and hence the magnon generation, is strongly suppressed since such a process does not satisfy energy conservation (up to exponentially small contributions). By contrast, for

biases $|eV_S| > E_Z$, the tunneling is efficient, resulting in significant magnon generation. Importantly, in this bias regime, *real* processes of creation of magnon states take place, such that the generated magnons remain in the bulk over a relatively long time scale. During their life-time, the magnons may experience inelastic scattering processes, which give rise to an equilibrium distribution of the bulk magnons. This distribution is characterized by an effective temperature of T, to be derived below. We assume that the inelastic magnon relaxation rate is much larger than the inverse dwell time of magnons in the quantum Hall bulk, i.e.,

$$\Gamma_{\text{in}} \gg \frac{\hbar}{t_{\text{dwell}}} \approx \min\left(\Gamma, \frac{\hbar v_m}{L_{\text{flight}}}\right). \tag{S5}$$

Here, v_m is the magnon velocity, and the typical length scale L_{flight} of the magnon propagation is the geometric size of the quantum Hall bulk. Under the assumption (S5), transitions between magnonic states are possible and thus the magnons lose coherence when they propagate before eventually decaying in one of the absorption regions. This loss of coherence permits us to treat the magnon generation or absorption regions independently of each other. This independence of tunneling processes in different regions is one of key assumptions of our theoretical analysis presented in detail below. Finally, the condition $\delta E < k_B T_0$ in Eq. (S4) is required in order to treat the magnonic spectrum as continuous.

S9.2. Model, electrical current, and noise. In this subsection, we consider a magnon generation and absorption process to take place in the vicinity of the contacts, **'A'**, **'B'**, **'C'**, **'D'**, **'E'**, and **'F'**, see Fig. 1(a) in the main text. By treating those regions as a single tunnel junction, we derive general formulas for the electrical current and noise generated in the junction. The main formulas will be used in subsection S9.3. Furthermore, the general formulas of the presented subsection can be straightforwardly extended to the case of a line junction (equilibrated regime), as is done in subsection S9.4, where the current and the noise in that regime are evaluated.

Each of the magnon generation and absorption regions consists of two co-propagating edge channels with opposite spins $s =\uparrow,\downarrow$. The Hamiltonian for these channels, the bulk magnons, and their coupling is $\hat{H} = \hat{H}_0 + \hat{H}_T + \hat{H}_m$, where $\hat{H}_0$ describes the kinetic energy for unbiased edge channels

$$\hat{H}_0 = -iv \sum_{s=\uparrow,\downarrow} \int dx \psi_s^\dagger(x) \partial_x \psi_s(x) \,. \tag{S6}$$

Here, $\psi_s^\dagger(x)$ creates an electron at position x with spin $s =\uparrow,\downarrow$ and both channels have velocity v. The bulk of the $\nu = 1$ quantum Hall states is spin-polarized with spin-$\uparrow$ electrons. The lowest-lying excitations in the bulk are spin excitations associated with spin flips ($\Delta S_z = \hbar$) called *magnons* [8]. The magnon excitation has a quadratic dispersion $\omega_{\boldsymbol{q}} \propto \boldsymbol{q}^2$ (with $\boldsymbol{q}$ the magnon momentum) and has an energy gap set by the Zeeman energy E_Z. The magnon Hamiltonian is thus

$$\hat{H}_m = \sum_{\boldsymbol{q}} (E_Z + \hbar\omega_{\boldsymbol{q}}) b_{\boldsymbol{q}}^\dagger b_{\boldsymbol{q}} \tag{S7}$$

in which $b_{\boldsymbol{q}}^\dagger$ ($b_{\boldsymbol{q}}$) creates (destroys) a magnon excitation with momentum $\boldsymbol{q}$. Due to conservation of angular momentum, electron tunneling from the inner edge channel with spin-$\downarrow$ to the outer one with spin-$\uparrow$ requires generation of magnons. This effect is captured by the tunneling Hamiltonian

$$\hat{H}_T = W\psi_\uparrow^\dagger(x=0)\psi_\downarrow(x=0,t)b^\dagger(x=0) + \text{h.c.}. \tag{S8}$$

Here, we assumed that tunneling only occurs at a single tunnel junction (taken at position $x = 0$); a generalization of H_T to the case of a line junction is straightforward. The amplitude for the tunneling is W. We next include the effect of the bias voltage V_S by introducing a time dependence in $H_T(t)$ through a gauge transformation [9]. This transformation leads to

$$\hat{H}_T(t) = W\psi_\uparrow^\dagger(0,t)\psi_\downarrow(0,t)b^\dagger(0,t)e^{-\frac{i}{\hbar}eV_St} + \text{h.c.}. \tag{S9}$$

Importantly, we have $eV_S > 0$ and $eV_S = 0$ describing the magnon generation and absorption regions, respectively. In other words, magnon generation is induced by a bias voltage (and in fact requires a sufficiently large voltage to overcome the magnon gap, as discussed above and will be explicit in calculation below). On the other hand, the absorption regions are unbiased.

From the Heisenberg equation of motion, we derive the tunneling current operator, given by

$$\hat{I}(t) = -\frac{ie}{\hbar}\left(W\psi_\uparrow^\dagger(0,t)\psi_\downarrow(0,t)b^\dagger(0,t)e^{-\frac{i}{\hbar}eV_St} - \text{h.c.}\right). \tag{S10}$$

We obtain the expectation value of the current at time t with the Keldysh technique: [9]

$$I(t) \equiv \langle\hat{I}(t)\rangle = \frac{1}{2}\sum_{\eta=\pm}\langle T_C\hat{I}(t^\eta)e^{-\frac{i}{\hbar}\int_C dt\hat{H}_T(t)}\rangle. \tag{S11}$$

Here, $\langle ...\rangle$ denotes averaging over the ground state of $H_0 + H_m$ and C denotes the Keldysh contour, consisting of a forward (backward) branch to evolve time from $-\infty(\infty)$ to $\infty(-\infty)$. The argument t^η denotes time t on the forward (backward) branch, parameterized by $\eta = \pm$. We assume that the tunneling is weak, $W \ll 1$, and thus keep terms only up to second order in the tunneling amplitude W. In this approximation, the expectation value of the current becomes

$$\begin{aligned}I(t=0) = -\frac{ie|W|^2}{2\hbar^2}\sum_{\eta,\eta_1=\pm1}\eta_1\int_{-\infty}^{\infty}dt_1\Big(&e^{\frac{i}{\hbar}eV_St_1}G_\uparrow^{\eta_1\eta}(t_1,0)G_\downarrow^{\eta\eta_1}(0,t_1)D^{\eta_1\eta}(t_1,0)\\ &- e^{-\frac{i}{\hbar}eV_St_1}G_\uparrow^{\eta\eta_1}(0,t_1)G_\downarrow^{\eta_1\eta}(t_1,0)D^{\eta\eta_1}(0,t_1)\Big).\end{aligned} \tag{S12}$$

Here, $G_s^{\eta_1\eta_2}(t_1,t_2) \equiv -i\langle T_C\psi_s(t_1^{\eta_1})\psi_s^\dagger(t_2^{\eta_2})\rangle$ is the fermionic Keldysh Green functions for the edge channel with spin $s =\uparrow,\downarrow$, while $D^{\eta_1\eta_2}(t_1,t_2) \equiv -i\langle T_Cb(t_1^{\eta_1})b^\dagger(t_2^{\eta_2})\rangle$ is the bosonic Keldysh Green functions for the magnons. By using the relation $G_s^{\eta_1\eta_2}(t_1,t_2) = -G_s^{\eta_2\eta_1}(t_2,t_1)$ (which follows from particle-hole

symmetry in the edge channel spectrum), Eq. (S12) reduces to

$$I(t=0) = -\frac{ie|W|^2}{2\hbar^2} \sum_{\eta,\eta_1=\pm 1} \eta_1 \int_{-\infty}^{\infty} dt_1 G_{\uparrow}^{\eta_1\eta}(t_1,0) G_{\downarrow}^{\eta\eta_1}(0,t_1) \\ \times \left(e^{\frac{i}{\hbar}eV_S t_1} D^{\eta_1\eta}(t_1,0) - e^{-\frac{i}{\hbar}eV_S t_1} D^{\eta\eta_1}(0,t_1) \right). \qquad \text{(S13)}$$

Since $G_s^{\eta\eta}(t)$ is an even function of t, the contribution from $\eta_1 = \eta$ vanishes and Eq. (S13) is further simplified as

$$\begin{aligned} I &= \frac{ie|W|^2}{\hbar^2} \int_{-\infty}^{\infty} dt_1 G_{\uparrow}^{>}(t_1) G_{\downarrow}^{<}(-t_1) \left(D^{>}(t_1) e^{\frac{i}{\hbar}eV_S t_1} - D^{<}(-t_1) e^{-\frac{i}{\hbar}eV_S t_1} \right) \\ &= \frac{ie|W|^2}{\hbar^2} \int \frac{d\omega_1}{2\pi} \int \frac{d\omega_2}{2\pi} \Big(G_{\uparrow}^{>}(\omega_1) G_{\downarrow}^{<}(\omega_2 - \frac{eV_S}{\hbar}) D^{>}(\omega_2 - \omega_1) \\ &\qquad - G_{\uparrow}^{<}(\omega_1) G_{\downarrow}^{>}(\omega_2 - \frac{eV_S}{\hbar}) D^{<}(\omega_2 - \omega_1) \Big). \end{aligned} \qquad \text{(S14)}$$

Equation (S14) is the central result of this subsection and will be used in subsection S9.3 for the tunnel-junction model and, with an appropriate modification, in subsection S9.4 for the line-junction model.

We now use the same formalism to obtain the zero frequency noise. The noise $S(t)$ is given by

$$\begin{aligned} S(t) &\equiv \langle \hat{I}(t)\hat{I}(0)\rangle + \langle \hat{I}(0)\hat{I}(t)\rangle - 2\langle \hat{I}(t)\rangle^2 \\ &= \sum_{\eta=\pm} \langle T_C \hat{I}(t^{\eta}) \hat{I}(0^{-\eta}) e^{-\frac{i}{\hbar}\int_C dt \hat{H}_T(t)} \rangle - 2\langle \hat{I}(t)\rangle^2. \end{aligned} \qquad \text{(S15)}$$

To second order in the tunneling strength, the zero frequency noise reads

$$\begin{aligned} S &= \int_{-\infty}^{\infty} dt S(t) \simeq \sum_{\eta=\pm} \int_{-\infty}^{\infty} dt \langle T_C \hat{I}(t^{\eta}) \hat{I}(0^{-\eta}) \rangle \\ &= \frac{2ie^2|W|^2}{\hbar^2} \int_{-\infty}^{\infty} dt e^{-\frac{i}{\hbar}eV_S t} (G_{\uparrow}^{>}(-t) G_{\downarrow}^{<}(t) D^{>}(-t) + G_{\uparrow}^{<}(-t) G_{\downarrow}^{>}(t) D^{<}(-t)) \\ &= \frac{2ie|W|^2}{\hbar^2} \int \frac{d\omega_1}{2\pi} \int \frac{d\omega_2}{2\pi} \Big(G_{\uparrow}^{>}(\omega_1) G_{\downarrow}^{<}(\omega_2 - \frac{eV_S}{\hbar}) D^{>}(\omega_2 - \omega_1) \\ &\qquad + G_{\uparrow}^{<}(\omega_1) G_{\downarrow}^{>}(\omega_2 - \frac{eV_S}{\hbar}) D^{<}(\omega_2 - \omega_1) \Big). \end{aligned} \qquad \text{(S16)}$$

By using the well-known expressions for the equilibrium Green functions of edge channels and magnons (both at temperature T_0), we obtain the following relation between the tunneling current I and the zero frequency noise S:

$$S = 2eI \coth\left(\frac{eV_S - \mu_m}{2T_0} \right). \qquad \text{(S17)}$$

Here, μ_m is the chemical potential of the magnons. Note that magnons have a non-zero chemical potential since we consider a non-equilibrium situation (finite voltage eV_S). Equation (S17) bears similarity to a usual formula for (zero-frequency) noise in tunnel junctions. The difference is in chemical potential μ_m entering Eq. (S17). This is because an electron tunneling in our problem involves creation or annihilation of a magnon.

S9.3. Current and noise in a single tunnel junction model. In this subsection, we consider a single tunnel junction model, which was described above. Within this model, we compute the electrical current and noise generated in the absorption regions by employing Eqs. (S14) and (S17). For generality, we allow for tunneling amplitude W to be different in the generation and absorption regions, as indicated by subscripts "ge" and "ab" below.

As discussed above, we treat the tunneling processes in the magnon generation and absorption regions independently of each other and thus calculate the electrical current in each region separately. Applying Eq. (S14), the electrical current in the magnon generation region is given as

$$\begin{aligned} I_{\text{ge}} = &\frac{2\pi}{\hbar} e|W_{\text{ge}}|^2 \rho_{\text{edge}}^2 \rho_m \int d\omega_1 \int d\omega_2 \theta(\omega_2 - \omega_1 - E_Z) \\ &\times \big[(1 - f(\omega_1)) f(\omega_2 - |eV_S|)(1 + b(\omega_2 - \omega_1 - \mu_m)) \\ &\quad - f(\omega_1)(1 - f(\omega_2 - |eV_S|)) b(\omega_2 - \omega_1 - \mu_m))\big]. \end{aligned} \tag{S18}$$

Here, we have used the equilibrium Green's functions for the edge channels, $G^{>}_{\uparrow/\downarrow}(\omega) = -2\pi i \rho_{\text{edge}}(1 - f(\omega))$ and $G^{<}_{\uparrow/\downarrow}(\omega) = 2\pi i \rho_{\text{edge}} f(\omega)$, as well as the Green's function for the magnons

$$\begin{aligned} D^{>}(\omega) &= -2\pi i \sum_{i_N, f_{N+1}} \sum_{\boldsymbol{q}} W_{i_N} \langle i_N | b_{\boldsymbol{q}} | f_{N+1} \rangle \langle f_{N+1} | b^\dagger_{\boldsymbol{q}} | i_N \rangle \delta(\omega - (E_Z + \omega_{\boldsymbol{q}})) \\ &= -2\pi i \sum_{\boldsymbol{q}} (1 + b(\omega_{\boldsymbol{q}} + E_Z - \mu_m)) \delta(\omega - (E_Z + \omega_{\boldsymbol{q}})) \\ &= -2\pi i \rho_m \theta(\omega - E_Z)(1 + b(\omega - \mu_m)). \end{aligned} \tag{S19}$$

The density of states ρ_{edge} and ρ_m for the edge modes and magnons are assumed to be energy independent. In Eq. (S19), $|i_N\rangle$ ($|f_N\rangle$) is an initial (final) Fock state with N magnons. In the second equality, we used that only states with $|f_{N+1}\rangle \propto b^\dagger_{\boldsymbol{q}} |i_N\rangle$ survive in the summation. Furthermore, W_{i_N} is the probability that the initial state is $|i_N\rangle$, and $\langle i_N | b_{\boldsymbol{q}} | f_{N+1} \rangle \langle f_{N+1} | b^\dagger_{\boldsymbol{q}} | i_N \rangle$ is further expressed as $\langle i_N | b_{\boldsymbol{q}} | f_{N+1} \rangle \langle f_{N+1} | b^\dagger_{\boldsymbol{q}} | i_N \rangle = (1 + b(\omega_{\boldsymbol{q}} + E_Z))$. This equality is based on that the magnon states follow a Bose distribution function $b(\omega - \mu_m) = 1/(e^{\beta(\omega - \mu_m)} - 1)$ with the magnonic chemical potential μ_m. The electrical current (S18) can now be written as

$$I_{\text{ge}} = -e(\Gamma^{\text{ge}}_{N+1,N} - \Gamma^{\text{ge}}_{N-1,N}), \tag{S20}$$

in terms of the rate $\Gamma^{\text{ge}}_{N+1,N}$ ($\Gamma^{\text{ge}}_{N-1,N}$) at which the magnon number increases (respectively, decreases) by one. Similarly, the current generated in the absorption regions can be written as

$$I_{\text{ab}} = -e(\Gamma^{\text{ab}}_{N+1,N} - \Gamma^{\text{ab}}_{N-1,N}). \tag{S21}$$

The rates in Eq. (S20) are given as

$$\begin{aligned}\Gamma^{\text{ge}}_{N+1,N} &= -\frac{2\pi}{\hbar}|W_{\text{ge}}|^2\rho^2_{\text{edge}}\rho_m \int d\omega_1 \int d\omega_2 \theta(\omega_2 - \omega_1 - E_Z) \\ &\times (1 - f(\omega_1))f(\omega_2 - |eV_S|)(1 + b(\omega_2 - \omega_1 - \mu_m)) \\ &= -\frac{2\pi}{\hbar}|W_{\text{ge}}|^2\rho^2_{\text{edge}}\rho_m \int d\omega(\omega - |eV_S|)(1 + b(\omega - \mu_m))b(\omega - |eV_S|)\theta(\omega - E_Z) \\ &= -\frac{\gamma_{\text{ge}}}{h}\int d\omega(\omega - |eV_S|)(1 + b(\omega - \mu_m))b(\omega - |eV_S|)\theta(\omega - E_Z),\end{aligned} \tag{S22}$$

and

$$\Gamma^{\text{ge}}_{N-1,N} = \frac{\gamma_{\text{ge}}}{h}\int d\omega(\omega - |eV_S|)b(\omega - \mu_m)b(|eV_S| - \omega)\theta(\omega - E_Z). \tag{S23}$$

Here, γ_{ge} is defined as $\gamma_{\text{ge}} \equiv (2\pi)^2|W_{\text{ge}}|^2\rho^2_{\text{edge}}\rho_m$ and is assumed to be constant in energy. Similarly, the rates in Eq. (S21) read

$$\begin{aligned}\Gamma^{\text{ab},i}_{N+1,N} &= -\frac{\gamma^{\text{ab},i}}{h}\int d\omega\omega(1 + b(\omega - \mu_m))b(\omega)\theta(\omega - E_Z), \\ \Gamma^{\text{ab},i}_{N-1,N} &= \frac{\gamma^{\text{ab},i}}{h}\int d\omega\omega b(\omega - \mu_m)b(-\omega)\theta(\omega - E_Z).\end{aligned} \tag{S24}$$

The superscript i labels here the five absorption regions shown in Figs. 1(a)-(b) in the main text. Combining Eqs. (S20)-(S24), we obtain the current generated in the magnon creation and absorption processes as

$$I_{\text{ge}} = \frac{e\gamma^{\text{ge}}}{h}\int_{E_Z}^{\infty} d\omega(\omega - |eV_S|)(b(\omega - |eV_S|) - b(\omega - \mu_m)) \tag{S25}$$

$$I^i_{\text{ab}} = \frac{e\gamma^{\text{ab},i}}{h}\int_{E_Z}^{\infty} d\omega\omega(b(\omega) - b(\omega - \mu_m)) \ . \tag{S26}$$

Note that these rates are independent of the number of magnons.

In the steady state, the number of magnons does not change in time and hence

$$I_{\text{ge}} = -\sum_{i=1}^{M} I^i_{\text{ab}} \tag{S27}$$

should hold. Here, $M = 5$ is the number of the absorption regions (see Figs. 1(a)-(b) in the main text). By using Eq. (S27), we can self-consistently determine the effective magnon chemical potential μ_m. We further assume that the magnon and the edge channels are all at the system base temperature T_0.

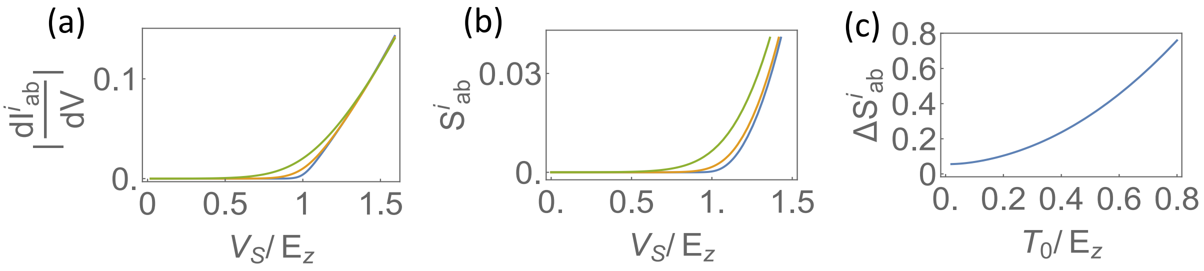

Supplementary Figure 9: Singe tunnel junction results: (a) Non-local differential conductance $\frac{dI^i_{\rm ab}}{dV}$ and (b) noise $S^i_{\rm ab}$ generated at an absorption region as a function of the bias voltage V at different temperatures T_0. Blue lines: $T_0 = 0.02E_Z$, orange lines: $T_0 = 0.05E_Z$, green lines: $T_0 = 0.1E_Z$, where E_Z is the Zeeman energy (magnon energy gap). (c) Excess noise $\Delta S^i_{\rm ab}$ vs temperature T_0 at fixed bias energy $eV_S = 1.5E_Z$. With increasing temperature, the excess noise monotonically increases as more magnons are generated, since a larger phase space becomes available at higher temperatures. Both differential conductance and noise are plotted in units of $\frac{e^2}{h}\gamma^{\rm ge}$. We set $e = k_B = 1$ for the plots.

The tunneling current generated in each individual absorption region is given by

$$I^i_{\rm ab} = \frac{\gamma^{{\rm ab},i}}{\gamma^{\rm ab}} \sum_{i=1}^{M} I^i_{\rm ab} = -\frac{\gamma^{{\rm ab},i}}{\gamma^{\rm ab}} I_{\rm ge}, \tag{S28}$$

where $\gamma^{\rm ab} \equiv \sum_{i=1}^{M} \gamma^{{\rm ab},i}$. At zero temperature, Eq. (S25) takes the simplified form

$$I_{\rm ge} \approx \frac{e\gamma^{\rm ge}}{2h}(|eV_S| - E_Z)^2\theta(|eV_S| - E_Z)\,, \tag{S29}$$

resulting in $I^i_{\rm ab}$ and the corresponding non-local conductance from Eq. (S28) as

$$I^i_{\rm ab} \approx -\frac{\gamma^{{\rm ab},i}}{\gamma^{\rm ab}} \frac{e\gamma^{\rm ge}}{2h}(|eV_S| - E_Z)^2\theta(|eV_S| - E_Z)\,, \tag{S30}$$

$$\frac{dI^i_{\rm ab}}{dV} \approx -\frac{\gamma^{{\rm ab},i}}{\gamma^{\rm ab}} \frac{e^2}{h}\gamma^{\rm ge}(|eV_S| - E_Z)\theta(|eV_S| - E_Z)\,. \tag{S31}$$

By using Eq. (S17), we obtain the noise generated at an absorption region

$$S^i_{\rm ab} = 2e\coth\left(\frac{\mu_m}{2T_0}\right)|I^i_{\rm ab}| \xrightarrow{\mu_m/T_0\to\infty} \frac{e^2}{h}\frac{\gamma^{{\rm ab},i}}{\gamma^{\rm ab}}\gamma^{\rm ge}(|eV_S| - E_Z)^2\theta(|eV_S| - E_Z)\,. \tag{S32}$$

By self-consistently calculating μ_m from the steady state condition Eq. (S27), we numerically obtain $I^i_{\rm ab}$, $\frac{dI^i_{\rm ab}}{dV}$, and $S^i_{\rm ab}$. We plot $\frac{dI^i_{\rm ab}}{dV}$, and $S^i_{\rm ab}$ as functions of the bias voltage at finite temperature in Supplementary Figs. 9(a)-(b). While the non-local conductance increases linearly in the bias voltage $|eV_S| - E_Z \gg T_0$, the noise increases quadratically. These characteristic behaviors of the non-local conductance and the noise are consistent with the zero temperature results, see Eqs. (S31) and (S32). The excess noise $\Delta S^i_{\rm ab}$ vs T_0 at

fixed bias is plotted in Supplementary Fig. 9(c). It is seen that the excess noise increases with increasing temperature T_0 in the single tunnel-junction model. This happens because more magnons are generated due to the larger magnonic phase space available at higher temperatures. This behavior is at variance with the experimental dependence of noise on temperature for a voltage $|eV_S|$ well above E_Z, see Fig. 3(e) in the main text. As we show below, at such voltages a different model—that of line junction corresponding to full equilibration—is applicable, which explains the experimental behavior of noise in Fig. 3(e).

S9.4. Current and noise in a line junction model. In contrast to the previous subsection, we assume here that magnon-generation and absorption processes occur along a line junction rather than in a single tunnel junction. We model such a line junction as an extended segment of length L with two co-propagating edge channels. Along this segment electrons tunnel incoherently between the edge channels along an array of tunnel junctions. The line junction is assumed to have a sufficiently large length L compared with the equilibration length ℓ_{eq} so that the edge channels and the magnons equilibrate fully. The equilibration continues until the net tunneling between the edge channels vanishes. While, in principle, one can imagine a situation in which the edge modes in this regime would be characterized by a non-trivial out-of-equilibrium distribution, we assume here that inelastic mechanisms allow the full system (the edge channels plus magnons) to relax to an equilibrium distribution. In the following, we use this line junction model to calculate the electrical current and noise generated in the magnon absorption regions.

We begin with the magnon generation process. As described above, the edge channels achieve full equilibration at positions $x \gg \ell_{\text{eq}}$, where the net tunneling current vanishes, i.e., $\langle I_{\text{ge}}(x)\rangle = 0$. We thus set

$$\begin{aligned}\langle I_{\text{ge}}(x)\rangle = \frac{2\pi}{\hbar} e|W_{\text{ge}}|^2 \rho_{\text{edge}}^2 \rho_m \int d\omega_1 \int d\omega_2 \theta(\omega_2 - \omega_1 - E_Z) \\ \times \big[(1 - f_\uparrow(\omega_1, x)) f_\downarrow(\omega_2, x)(1 + b(\omega_2 - \omega_1 - \mu_m)) \\ - f_\uparrow(\omega_1, x)(1 - f_\downarrow(\omega_2, x)) b(\omega_2 - \omega_1 - \mu_m)\big] = 0. \end{aligned} \tag{S33}$$

Here, we assumed that the magnons and the edge channels have relaxed to individual equilibrium distribution functions f_s and b and used the corresponding generalization of Eq. (S14). The simplest solution for distribution functions that satisfy Eq. (S33) is that the edge channels and the magnon system acquire the same temperature T ("true equilibration"), i.e.,

$$f_{\uparrow/\downarrow}(\omega) = \frac{1}{e^{(\omega - \mu^{\text{ge}}_{\uparrow/\downarrow})/T} + 1}, \quad b(\omega) = \frac{1}{e^{(\omega - \mu_m)/T} - 1}, \tag{S34}$$

$$\mu^{\text{ge}}_\downarrow - \mu^{\text{ge}}_\uparrow = \mu_m\,. \tag{S35}$$

However, for a low temperature and a reasonable size of the line junction L, this true equilbration will not be reached. Indeed, the equilibration process becomes strongly suppressed once the chemical potential difference between the edge channels reduces down to E_Z,

$$\mu^{\text{ge}}_\downarrow - \mu^{\text{ge}}_\uparrow = E_Z\,. \tag{S36}$$

A further magnon generation would correspond to $\mu_{\downarrow}^{\text{ge}} - \mu_{\uparrow}^{\text{ge}}$ becoming smaller than E_Z and is strongly suppressed with an exponential factor $e^{(\mu_{\downarrow}^{\text{ge}} - \mu_{\uparrow}^{\text{ge}} - E_Z)/T}$. Therefore, for a realistic (not exponentially large) length L of the line junction, the equilibration effectively stops at the saturation point (S36). While this is a kind of "pre-equilibration", we term this situation "full equilibration" for brevity. Correspondingly, the case of a short junction, when resulting electrochemical potentials do not reach Eq. (S36), i.e., $\mu_{\downarrow}^{\text{ge}} - \mu_{\uparrow}^{\text{ge}} > E_Z$ is termed in this paper "partial equilibration".

From charge conservation, we can relate the electrochemical potential of the edge channels emanating from the source contact with $\mu_{\uparrow/\downarrow}^{\text{ge}}$ as

$$\mu_{\downarrow}^{\text{ge}} + \mu_{\uparrow}^{\text{ge}} = |eV_S|\,. \tag{S37}$$

Similarly, we obtain from charge conservation an equation for the absorption regions

$$\mu_{\downarrow}^{\text{ab},i} + \mu_{\uparrow}^{\text{ab},i} = 0. \tag{S38}$$

Here, $\mu_{\downarrow,\uparrow}^{\text{ab},i}$ are the electrochemical potentials after the magnon absorption process in each individual magnon-absorption region. The electrochemical potentials $\mu_{\uparrow,\downarrow}^{\text{ab},i}$ in the absorption region are related to the electrochemical potentials $\mu_{\uparrow,\downarrow}^{\text{ge}}$ in the generation region by the steady state condition

$$I_{\text{ge}} = -\sum_{i=1}^{M} I_{\text{ab}}^{i}, \tag{S39}$$

where M is the number of absorption regions. Equation (S39) guarantees that in the steady state the magnon generation and absorption rates are identical, keeping the number of magnons in the bulk constant. Combining Eqs. (S36)-(S39), we thus obtain

$$\mu_{\downarrow}^{\text{ge}} = \frac{|eV_S| + E_Z}{2}, \quad \mu_{\uparrow}^{\text{ge}} = \frac{|eV_S| - E_Z}{2}, \quad \mu_{\downarrow}^{\text{ab}} = \frac{|eV_S| - E_Z}{2M}, \quad \mu_{\uparrow}^{\text{ab}} = -\frac{|eV_S| - E_Z}{2M}. \tag{S40}$$

Here, we assumed for simplicity that in each individual absoprion region, the magnons are absorbed with equal probabilities, and hence $\mu_{\downarrow}^{\text{ab},i} = \mu_{\downarrow}^{\text{ab}}$ and $\mu_{\uparrow}^{\text{ab},i} = \mu_{\uparrow}^{\text{ab}}$. We further find an equation for energy conservation, which reads for our experimental geometry (shown in Fig. 1(a) in the main text)

$$\begin{aligned} &2 \times \frac{(eV_S)^2}{2h} + 6 \times \frac{\pi^2 T_0^2}{6h} \\ &= \frac{(|eV_S| + 2\mu_{\uparrow}^{\text{ab}})^2 + (2\mu_{\downarrow}^{\text{ab}})^2 + \sum_{s=\uparrow,\downarrow}((\mu_s^{\text{ge}} + \mu_s^{\text{ab}})^2 + (2\mu_s^{\text{ab}})^2)}{2h} + 6 \times \frac{\pi^2 T^2}{6h}\,. \end{aligned} \tag{S41}$$

Here, we used the fact that, in our geometry, there are in total six edge channels and out of them two channels are biased. Further, T_0 is the ambient temperature of the contacts and T is the effective temperature of the system as a result of equilibration. We assume that the edge modes reach equilibrium with the entire system sharing the same temperature T. We extract this temperature from Eq. (S40) and (S41) and it reads

$$T = \sqrt{T_0^2 + \frac{3(|eV_S| - E_Z)(2E_Z + 3|eV_S|)}{(5\pi)^2}\theta(|eV_S| - E_Z)}. \tag{S42}$$

As derived in the next subsection, the total noise in the individual absorption region is given by

$$S^i = \frac{e^2}{h} k_B (T + T_0) \tag{S43}$$

and thus the corresponding excess noise is given by

$$S_I^i = \frac{e^2}{h} k_B (T_0 + T) - 2\frac{e^2}{h} k_B T_0 = \frac{e^2}{h} k_B (T - T_0). \tag{S44}$$

The superscript i denotes the five absorption regions. The tunneling current and the corresponding differential non-local conductance at an absorption region are given by

$$I_{\text{ab}} = \frac{e}{2Mh}\theta(|eV_S| - E_Z)(|eV_S| - E_Z), \tag{S45}$$

$$\frac{dI_{\text{ab}}}{dV} = \frac{e^2}{2Mh}\theta(|eV_S| - E_Z). \tag{S46}$$

Note that temperature corrections to the dc current are exponentially small (cf. Eq. (S36) and the following discussion). By contrast, the noise is linearly dependent on the effective temperature.

We can define an effective Fano factor in the individual absorption region as $F_{\text{ab}} \equiv S_I/(2eI_{\text{ab}})$. Using that $M = 5$ in our geometry, we find that, in the strong bias regime ($|eV_S| \gg E_Z, T_0$), the Fano factor approaches

$$F_{\text{ab}} \to \frac{3}{\pi} \approx 0.95. \tag{S47}$$

The measured current and the excess noise are given by

$$I = \frac{\langle I_{\text{ab}}^{\mathbf{B}} \rangle - \langle I_{\text{ab}}^{\mathbf{D}} \rangle}{2}, \quad S_I = \frac{S_I^{\mathbf{B}} + S_I^{\mathbf{D}}}{4}. \tag{S48}$$

Importantly, the factor of 1/2 entering the current and the factor 1/4 for the excess noise come from the experimental geometry for measuring these quantities, see Fig. 1(a) in the main text and Supplementary Fig. 6(a). More specifically, the floating contact in these figures emits currents and fluctuations on two edges of the device. The contact measuring the floating-contact electrochemical potential μ_{FC} and its fluctuations $\delta\mu_{\text{FC}}$ is located on one of these edges and thus receives only half of the emission. We have also taken into account that two absorption regions **'B'** and **'D'** contribute to the measured noise and the measured current.

The non-local conductance and the noise in the line junction model display eV_S-characteristics distinct from those in the single tunnel junction model. While the non-local conductance in the single tunnel junction is linear in eV_S, the line junction model has a constant differential conductance, see Eq. (S46). Further, the noise at large bias increases quadratically with eV_S in the single tunnel junction, whereas it grows linearly as a function of eV_S in the line junction model, see Eqs. (S44) and (S42). We plot the calculated excess noise vs eV_S at a few different temperatures in Supplementary Fig. 10(a). At low temperatures,

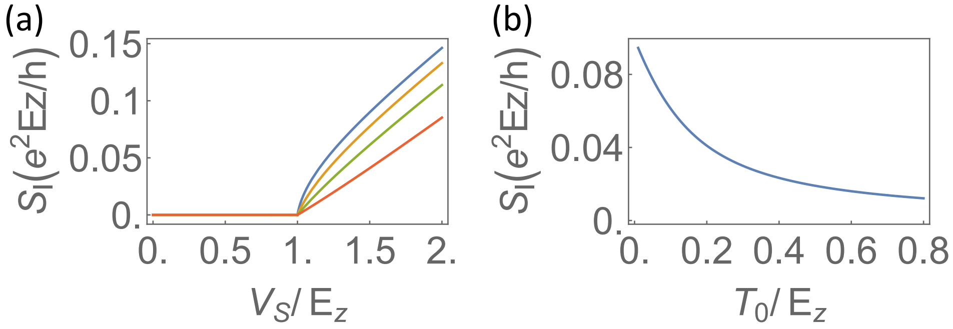

Supplementary Figure 10: Excess noise in the line junction model (a) as function of the bias energy eV_S at different temperatures (blue: $T_0 = 0.02E_Z$, orange: $T_0 = 0.05E_Z$, green: $T_0 = 0.1E_Z$, red: $T_0 = 0.2E_Z$) and (b) as a function of temperature T_0 at fixed bias voltage $eV_S = 1.5E_Z$. We set $e = k_B = 1$ for these plots.

$T_0 \to 0$, we find $S_I \propto \sqrt{|eV_S| - E_Z}\theta(|eV_S| - E_Z)$ in the vicinity of $|eV_S| = E_Z$. The cusp-like singularity at $|eV_S| = E_Z$ is an artifact of the full-equilibration assumption, which, for fixed junction length L, in fact ceases to be applicable when $|eV_S|$ approaches E_Z, see next subsection. We also plot the temperature dependence of the excess noise at fixed voltage eV_S in Supplementary Fig. 10(b). We see that the excess noise decreases with increasing temperature, which is in full consistency with the experimental observation presented in Fig. 3(e) in the main text.

S9.5. Noise generated in a line junction. In this subsection, we present a derivation of the formula (S43) (equivalently, Eq. (1) in the main text) for the noise of the tunneling current generated in the magnon absorption regions. A scheme of the line junction formed by two co-propagating edges in an absorption region is shown in Supplementary Fig. 11. In such a region, electron tunneling between the two edge channels is only possible by absorption of impinging magnons, in view of the angular momentum conservation constraint. We assume that the line junction consists of a series of tunnel junctions. Under the assumption of full equilibration of the edge channels, we show that the noise is dominantly generated at the downstream end of the line junction (indicated by the yellow circle in Fig. 11). The magnitude of noise is governed by the effective temperature in this "noise spot". This noise-generating mechanism, which is described in more detail below, is essentially the same as that identified and analyzed in Refs. [10] and [11] in the context of fractional quantum Hall edges.

We begin by investigating a small segment of the line junction which contains only a single tunnel junction. Charge conservation relates the two channel charge currents as

$$I_{j,\uparrow} = I_{j-1,\uparrow} - I_{\text{tun},j}, \quad I_{j,\downarrow} = I_{j-1,\downarrow} + I_{\text{tun},j}. \tag{S49}$$

Here, $I_{j,\uparrow/\downarrow}$ is the charge current flowing along segment j ($1 \leq j \leq N$) of the edge channel with spin $\uparrow$ / $\downarrow$,

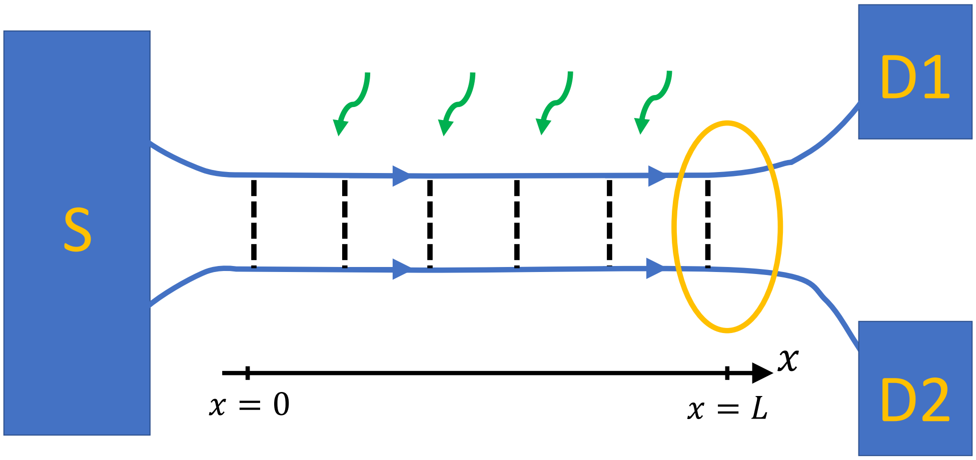

Supplementary Figure 11: Schematic plot of a line junction in a magnon-absorption region. The electron tunneling between two co-propagating edge modes is allowed by absorbing magnons (indicated by green arrows). The noise of the total tunneling current is mostly generated in the vicinity of $x = L$ ("noise spot" shown by a yellow circle).

$I_{\text{tun},j}$ represents the tunneling current in tunnel junction j, and the fluctuations in $I_{\text{tun},j}$ is decomposed as

$$\delta I_{\text{tun},j} = g_j \frac{e^2}{h}(\delta V_{j-1,\uparrow} - \delta V_{j-1,\downarrow}) + \delta I^{\text{int}}_{\text{tun},j} = g_j(\delta I_{j-1,\uparrow} - \delta I_{j-1,\downarrow}) + \delta I^{\text{int}}_{\text{tun},j}. \tag{S50}$$

The first contribution in Eq. (S50) comes from the fluctuations currents impinging (from the left) on the tunnel junction. The second contribution describes intrinsic current fluctuation generated in the junction. This term includes contributions of local-equilibrium (i.e., thermal) and out-of-equilibrium (i.e., shot-noise) origin. Finally, g_j is the tunneling probability, which generically depends on the strength of the local tunneling current and how the incoming states occupy microscopic states. We assume g_j to be weak: $g_j \ll 1$. By using Eqs. (S49) and (S50), we can relate the currents in each segment according to

$$\begin{pmatrix} \delta I_{j,\uparrow} \\ \delta I_{j,\downarrow} \end{pmatrix} = M_j \begin{pmatrix} \delta I_{j-1,\uparrow} \\ \delta I_{j-1,\downarrow} \end{pmatrix} + \boldsymbol{v}_j, \tag{S51}$$

with the matrix M_j and the vector $\boldsymbol{v}_j$ are given by

$$M_j = \begin{pmatrix} 1-g_j & g_j \\ g_j & 1-g_j \end{pmatrix}, \quad \boldsymbol{v}_j = \delta I^{\text{int}}_{\text{tun},j} \begin{pmatrix} -1 \\ 1 \end{pmatrix}. \tag{S52}$$

Equation (S51) can be solved recursively with the solution

$$\begin{pmatrix} \delta I_{N,\uparrow} \\ \delta I_{N,\downarrow} \end{pmatrix} = (\prod_{j=1}^{N} M_{N-j+1}) \begin{pmatrix} \delta I_{0,\uparrow} \\ \delta I_{0,\downarrow} \end{pmatrix} + \sum_{j=1}^{N} \left(\prod_{j'=1}^{N-j} M_{N-j'+1} \right) \boldsymbol{v}_j. \tag{S53}$$

In the limit $\sum_{j=1}^{N} g_j \gg 1$, we can neglect term exponentially suppressed in N so that Eq. (S53) reduces to

$$\begin{pmatrix} \delta I_{N,\uparrow} \\ \delta I_{N,\downarrow} \end{pmatrix} \approx \frac{\delta I_{\uparrow}^{0} + \delta I_{\downarrow}^{0}}{2} \begin{pmatrix} 1 \\ 1 \end{pmatrix} - \sum_{j=1}^{N} e^{-2\sum_{j'=j+1}^{N} g_{j'}} \delta I_{\text{tun},j}^{\text{int}} \begin{pmatrix} 1 \\ -1 \end{pmatrix}. \tag{S54}$$

Next, we assume uniform tunneling, i.e., $g_j = g$, and take the continuum limit (justified by large N and small g). We then obtain the noise measured in drains $D1$ and $D2$ as

$$S_{D1} = S_{D2} = \overline{(\delta I_{N,\uparrow})^2} = \overline{(\delta I_{N,\downarrow})^2} = \frac{e^2}{h} T_0 + \frac{1}{a} \int_0^L dx e^{-4g\frac{L-x}{a}} \overline{(\delta I_{\text{tun}}^{\text{int}}(x))^2}. \tag{S55}$$

Here, $\overline{(\delta I_{N,s})^2} \equiv \int dt (\overline{\delta I_{N,s}(t)\delta I_{N,s}(0)} + \overline{\delta I_{N,s}(0)\delta I_{N,s}(t)})$ denotes the zero frequency noise of $\delta I_{N,s}(t)$ with $s = \uparrow, \downarrow$, a is the distance between two consecutive tunnel junctions, and L is the size of the line junction, i.e., $L = Na$. In Eq. (S55), we have used the fact that the intrinsic tunneling currents from different junctions are not correlated to each other. Note also that the exponential factor in the integrand shows explicitly that the noise is dominantly generated in the vicinity of $x = L$ ("noise spot") in the equilibrated regime, $L \gg \ell_{\text{eq}}$, where $\ell_{\text{eq}} = a/4g$ is the equilibration length. In the noise spot, the two edge modes will be fully equilibrated such that $\mu_m = \mu_\downarrow - \mu_\uparrow$, and thus the equilibrium noise dominates over the shot noise contribution to $\overline{(\delta I_{\text{tun}}^{\text{int}}(x))^2}$. We may therefore approximate $\overline{(\delta I_{\text{tun}}^{\text{int}}(x))^2}$ as

$$\overline{(\delta I_{\text{tun}}^{\text{int}}(x))^2} \approx 4g \frac{e^2}{h} k_B T, \tag{S56}$$

where T is the local temperature of the edge channels after equilibration. By inserting the local noise (S56) into Eq. (S55) and performing the integral, we arrive in the limit of full equilibration, $L/\ell_{\text{eq}} = 4Lg/a \gg 1$, at our final expression

$$S_{D1} = S_{D2} = \frac{e^2}{h} k_B (T + T_0). \tag{S57}$$

This completes the derivation of Eq. (S43) of the preceding section.

S9.6. Voltage dependence of equilibration length, overall behavior of the noise, and comparison to experimental data. In this subsection, we calculate the dependence of the equilibration length on the bias voltage $|eV_S|$. This allows us to demonstrate that the single tunnel junction model is applicable for $|eV_S|$ sufficiently close to E_Z, while the line junction model works for larger $|eV_S|$. We then compare the theoretical predictions with the experimental data.

To investigate the bias voltage dependence of the equilibration length ℓ_{eq} in the magnon generation region, we consider a series of N' tunnel junctions, neglecting possible quantum-interference effects between them. The tunneling currents in the individual tunnel junction are then added up and contribute the total tunnel current as $I_{\text{ge}}^{\text{tot}} = \sum_{j=1}^{N'} I_{\text{ge}}^{j}$. By using the expression for the tunneling current at a single tunnel junction, Eq. (S29), we arrive at

$$I_{\text{ge}}^{\text{tot}} = \frac{e}{2h} N' \gamma^{\text{ge}} (|eV_S| - E_Z)^2 \theta(|eV_S| - E_Z), \tag{S58}$$

where we assumed that γ^{ge} is the same for each tunnel junction. This result is valid as long as N' is not too large, so that the line junction effectively works as a tunnel junction with $\gamma^{\text{ge}} \to N'\gamma^{\text{ge}}$. The equilibration length is thus obtained as $\ell_{\text{eq}} = N'a$ (with a being the distance between the adjacent junctions) at which $I_{\text{ge}}^{\text{tot}}$ is equal to the total tunneling current $\frac{e}{2h}(|eV_S| - E_Z)$ by the full equilibration, i.e.,

$$I_{\text{ge}}^{\text{tot}} = \frac{e}{2h}N'\gamma^{\text{ge}}(|eV_S| - E_Z)^2\theta(|eV_S| - E_Z) = \frac{e}{2h}(|eV_S| - E_Z)\theta(|eV_S| - E_Z). \quad \text{(S59)}$$

From this equation, we obtain an expression for ℓ_{eq} as

$$\ell_{\text{eq}} = N'a = \frac{a}{\gamma^{\text{ge}}(|eV_S| - E_Z)} \equiv \frac{1}{\gamma(|eV_S| - E_Z)}. \quad \text{(S60)}$$

Here, $\gamma \equiv \gamma^{\text{ge}}/a$ is a parameter associated with the tunneling strength in the tunnel junctions. When the voltage $|eV_S| > E_Z$ approaches E_Z, the equilibration length diverges and thus prevents the edge channels from reaching the full equilibration. Thus, there is an intermediate regime of $|eV_S|$ slightly exceeding E_Z that can not be captured by full equilibration of the line junction model considered in the previous subsection, but is rather effectively described by the single tunnel junction model. This leads in particular to a smearing of the cusp-like behavior of noise at $|eV_S| = E_Z$ obtained in the line junction model.

Based on the bias voltage dependence of ℓ_{eq} calculated above [see Eq. (S60)], we thus identify the following three regimes, see Supplementary Figure 12: (i) Biases $|eV_S| < E_Z$ result in no magnon generation and thus no excess noise (discarding exponentially small contributions). (ii) In a narrow region $0 < |eV_S| - E_Z < \frac{1}{\gamma L}$ (which corresponds to $\ell_{\text{eq}} \equiv \frac{1}{\gamma(|eV_S| - E_Z)} > L$), the equilibration in magnon absorption and generation regions is only partial, as in the single tunnel junction model. In this regime, the noise generation is of non-equilibrium nature, resulting in $S_I \propto (|eV_S| - E_Z)^2$, see Eq. (S32). (iii) For larger biases $|eV_S| > E_Z + \frac{1}{\gamma L}$ and hence $\ell_{\text{eq}} < L$, the edge channels and magnons achieve full equilibration in the magnon absorption and generation regions.

In Supplementary Figure 12, these theoretical predictions are compared to typical experimental data. The green and red line show the theoretical result for the single tunnel junction and line junction models that are applicable in the regimes (ii) and (iii), respectively. These predictions are in a good agreement with experimental observations. Furthermore, we find that for $|eV_S|$ sufficiently exceeding E_Z [regime (iii)], the line junction model correctly reproduces several experimental observations: (1) a rather sharp increase, followed by a saturation, of the non-local conductance as a function of the bias voltage [compare Fig. 1(f) in the main text with Eq. (S46)], (2) a linear behavior of the noise as a function of the bias voltage [compare Fig. 2(d) with Supplementary Fig. 10(a)], and (3) the temperature dependence of the excess noise [compare Fig. 3(e) with Supplementary Fig. 10(b)].

References

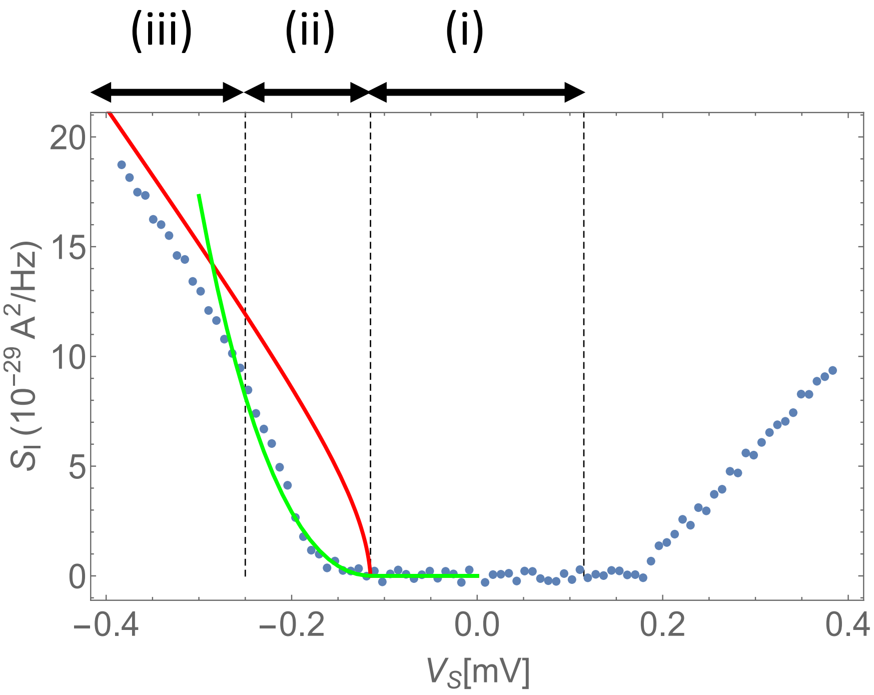

Supplementary Figure 12: Three different regimes of the noise S vs the bias voltage V_S: (i) regime of no magnon generation, (ii) partially equilibrated regime, and (iii) fully equilibrated regime. While the single tunnel junction model (green line) describes regime (ii), regime (iii) is captured by the line junction model (red line). The blue dots represent experimental data (the same set as in Fig. 3(b) in the main text). The value of γ in regime (ii) is chosen from a fit of the experimental data to Eqs. (S58) and (S60), which yields a dimensionless parameter $\gamma E_Z L = 0.8$.

1. Purdie, D. *et al.* Cleaning interfaces in layered materials heterostructures. *Nature communications* **9**, 5387 (2018).

2. Pizzocchero, F. *et al.* The hot pick-up technique for batch assembly of van der Waals heterostructures. *Nature communications* **7**, 11894 (2016).

3. Wang, L. *et al.* One-dimensional electrical contact to a two-dimensional material. *Science* **342**, 614–617 (2013).

4. Hunt, B. *et al.* Direct measurement of discrete valley and orbital quantum numbers in bilayer graphene. *Nature communications* **8**, 948 (2017).

5. Zibrov, A. A. *et al.* Tunable interacting composite fermion phases in a half-filled bilayer-graphene Landau level. *Nature* **549**, 360–364 (2017).

6. Kumar, R. *et al.* Observation of ballistic upstream modes at fractional quantum hall edges of graphene. *Nature communications* **13**, 213 (2022).

7. Srivastav, S. K. *et al.* Determination of topological edge quantum numbers of fractional quantum hall phases by thermal conductance measurements. *Nature Communications* **13**, 5185 (2022).

8. Girvin, S. M. The quantum Hall effect: Novel excitations and broken symmetries. In Comtet, A., Jolicœur, T., Ouvry, S. & David, F. (eds.) *Topological aspects of low dimensional systems*, 53–175 (Springer Berlin Heidelberg, Berlin, Heidelberg, 1999).

9. Martin, T. Noise in mesoscopic physics. In Bouchiat, H., Gefen, Y., Guéron, S., Montambaux, G. & Dalibard, J. (eds.) *Proceedings of the Les Houches Summer School, Session LXXXI* (Elsevier, New York, 2005).

10. Park, J., Mirlin, A. D., Rosenow, B. & Gefen, Y. Noise on complex quantum Hall edges: Chiral anomaly and heat diffusion. *Phys. Rev. B* **99**, 161302 (2019).

11. Spånslätt, C., Park, J., Gefen, Y. & Mirlin, A. D. Topological classification of shot noise on fractional quantum Hall edges. *Phys. Rev. Lett.* **123**, 137701 (2019).
